# A Gentleman in Disguise

**Published:** 1875-08

**Authors:** 


### A GENTLEMAN IN DISGUISE.

We wish to give some sort of lasting immortality to the name of James Siberson, a conductor on the Watervliet Turnpike and Railroad Company, of Albany. We took the horse-car, over the destinies of which he presides, at the steamboat landing in Albany the other morning, and on entering requested him to let us off at the Delevan House. Ten minutes later he approached, saying, "I forgot to speak to you. We are three blocks past the Delevan now. Here's your fare back." A car was passing in the other direction, which he stopped and transferred us to. We were so astounded at this remarkable civility, and it was all over so quickly, that we had no chance to decline or discuss the offer. But we learned the name of the gentleman in the guise of a conductor, and determined to do him a service if it ever fell in our way. If James Siberson ever needs help let him apply to us. If we ever own a horse-railroad we shall invite James Siberson to be its superintendent, if he proves to be as good a man as we think he is.

